# Day workers suffering from a wider range of sleep problems are more likely to experience suicidality

**DOI:** 10.1007/s41105-016-0067-5

**Published:** 2016-06-23

**Authors:** Yuuki Matsumoto, Naohisa Uchimura, Tetsuya Ishida, Kouji Toyomasu, Yoshitaka Morimatsu, Mihoko Mori, Nanae Kushino, Michiko Hoshiko, Tatsuya Ishitake

**Affiliations:** 1 Department of Environmental Medicine, Kurume University School of Medicine, 67 Asahi-machi, Kurume, 830-0011 Japan; 2 Department of Neuropsychiatry, Kurume University School of Medicine, Kurume, Japan; 3 Institute of Health and Sports Science, Kurume University, Kurume, Japan

**Keywords:** Depression, Sleep, Sleep deprivation, Suicidal ideation, Suicide

## Abstract

Both a higher suicide rate and widespread sleep problems are serious health concerns in Japan when compared with those of other countries. We investigated the relationship between suicidal ideation and sleep problems in Japanese day workers using the 3-dimensional sleep scale (3DSS), which measures three sleep elements (phase, quality, and quantity). Data from 635 Japanese day workers (461 mens and 174 womens) were included. The 3DSS was used to assess participants’ sleep condition. Participants were classified into eight sleep types based on scores of phase, quality, and quantity: All Good Sleep, Owl (poor phase), Inefficient (poor quality), Short (poor quantity), Owl + Inefficient (poor phase and quality), Owl + Short (poor phase and quantity), Inefficient + Short (poor quality and quantity), and All Poor Sleep. We assessed participants’ suicidal ideation using question 19 of the self-rating depression scale (SDS); 119 cases (18.7 %) had ratings of 2–4 for this question and were considered to have suicidal ideation. The higher the number of sleep problems, the higher the risk of suicidal ideation compared to sleep types not indicative of problems. All Poor Sleep had the highest risk of the eight sleep types. Individuals with Owl + Short, Inefficient + Short, or All Poor Sleep had a significant risk of suicidal ideation even after adjusting for hopelessness and nightmares. Our findings suggested that sleep problems assessed by the 3DSS were related to suicidal ideation. Analysis of various aspects of sleep could be helpful for suicide prevention.

**Electronic supplementary material:**

The online version of this article (doi:10.1007/s41105-016-0067-5) contains supplementary material, which is available to authorized users.

## Introduction

Previous studies have reported the relationship between suicide and sleep problems [1–4]; both are very serious health concerns in Japan. It is well known that many Japanese people committed suicide in 1998 [5]. Major Japanese banks were affected by financial panic around that time, which may have caused the rapidly increased suicide rate. The peak number of suicides occurred in 2003 (34,427), and the number has since gradually declined, with the number of suicides in 2014 returning to nearly that of the pre-1998 era. However, the suicide rate of young individuals has remained high, while that of the aged has declined substantially. Unfortunately, the largest cause of deaths among those aged 15–39 in Japan is suicide, which differs from other developed nations [5]. In addition, Japanese individuals have sleep problems, which are worse than those of other countries [6]. Since Japan is a 24-h society and employees are likely to work long hours, the nation is famous for eveningness and lack of sleep. Therefore, we considered the chronic high rate of Japanese workers’ suicides to be related to their sleep problems, and deemed it a relationship in need of investigation.

It is difficult to assess sleep conditions in a 24-h nation, because sleep phase problems (i.e., sleep regularity and chronotype) as well as sleep quality (i.e., sleep efficiency and sleep satisfaction) and sleep quantity (i.e., sleep duration and sleep adequacy) must be considered. To this end, we developed a scale to measure the three elements of sleep (phase, quality, and quantity) [7, 8], which we named the “3-dimensional sleep scale” or 3DSS. The 3DSS can be used to classify participants by sleep type based on their scale scores. Previous studies have reported that each sleep element could relate to mental disorders independently [9–11], and signs and symptoms such as depression or suicidal ideation always precede actual suicide [12]. Thus, sleep phase, quality, and quantity should be assessed separately.

The present study was conducted to verify the relationship between the process of suicide and sleep problems in Japanese workers using the 3DSS.

## Methods

### Participants

Data were collected in June 2013 [7], with the cooperation of three companies in Japan and collaborators working as industrial physicians. We gave participants the questionnaires at their workplace and collected them later that day. Eligible participants were 746 Japanese employees and 721 (96.7 %) responded. Shift workers and respondents with missing data (86) were excluded. Finally, we included data from 635 day workers (461 males and 174 females) for analysis in this study. The age (mean ± SD) of the participants was 40.5 ± 8.6 years (male: 41.1 ± 8.9 years; female: 38.7 ± 7.6 years).

### Measures

**Fig. 1:**
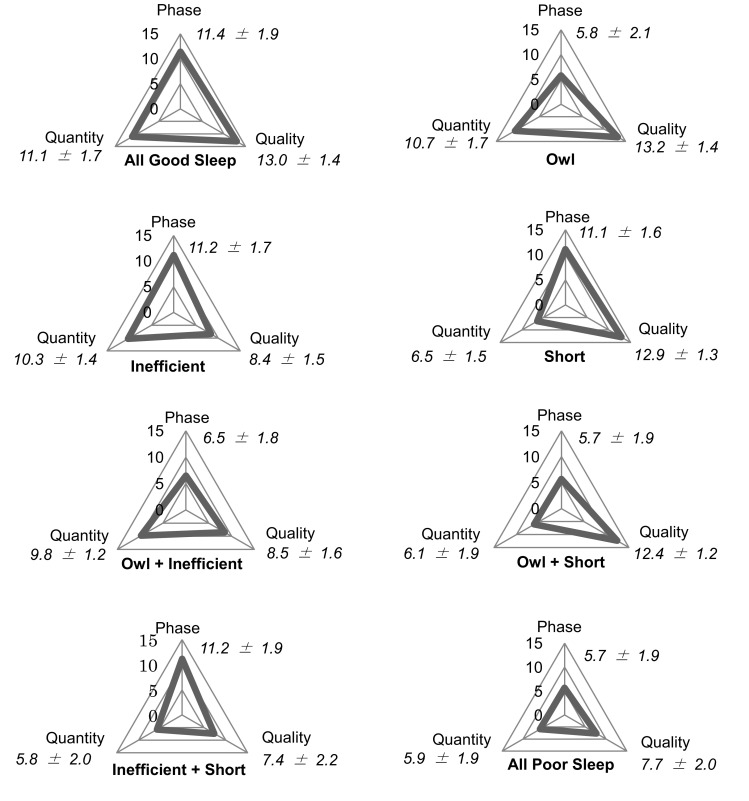
Displaying 3-dimensional sleep conditions of the eight sleep types and 3DSS scores (mean ± standard deviation)

We used the 3DSS as a sleep scale. It is designed for use with Japanese day workers and its reliability and validity have been established [7]. The 3DSS consists of three categories (phase, quality, and quantity). All questions ask about usual sleep habits in the past month. Each category consists of five questions, for a total of 15 questions. Respondents choose a response option that fits their sleep habits from among four choices (questions, responses, and scoring methods are presented in the Supplementary Material). The range of each category’s score is 0–15; the higher the score, the better the sleep status. The cut-off value for phase or quantity scores is 8/9, and that of quality scores is 10/11 [8]. Table 1 and Fig. 1 displays the eight sleep types based on cut-off points.

**Table 1:** Number of participants belonging to the eight sleep types based on the cut-off points of the 3-dimensional sleep scale (3DSS)

Sleep type	*n* (%)	Relation to 3DSS cut-off points		
		Phase (8/9)	Quality (10/11)	Quantity (8/9)
All Good Sleep	122 (19.2)	H	H	H
Owl	70 (11.0)	L	H	H
Inefficient	61 (9.6)	H	L	H
Short	61 (9.6)	H	H	L
Owl + Inefficient	37 (5.8)	L	L	H
Owl + Short	85 (13.4)	L	H	L
Inefficient + Short	109 (17.2)	H	L	L
All Poor Sleep	90 (14.2)	L	L	L

*H* higher than cut-off point, *L* lower than cut-off point

The self-rating depression scale (SDS) is an established scale to measure depression and its reliability and validity have been established [13–15]. It consists of 20 questions about appetite, sexual desire, sleep, physical complaints, hopefulness, suicidal ideation, and so on. Questions are answered by selecting one of the four response options: (1) never or a little of the time, (2) some of the time, (3) a good part of the time, (4) most of the time. The higher the score, the more serious the depression. Participants were classified into three groups based on the following cut-off points: normal ≤39, slight depression = 40–49, moderate depression ≥50.

To evaluate suicidal ideation, we used Question 19 of the SDS: “I feel that others would be better off if I were dead.” Mizuno demonstrated a risk of suicide for Japanese individuals who selected a response choice of (2), (3), or (4) for this question [16]. Therefore, we identified participants who chose response options (2)–(4) for Question 19 as having suicidal ideation, and those who chose (1) as not having suicidal ideation. Furthermore, it is important to consider how depression affects suicidal ideation when both appear at the same time. Fried described suicidal ideation as being associated with specific rather than overall symptoms of depression, such as hopelessness, nightmares, and insomnia [17]. Thus, in this study, we used hopelessness and nightmares as adjustment factors instead of overall depression. Question 14 of the SDS, “I feel hopeful about the future,” was used as an indicator of hopelessness, and participants who chose response option (1) for this question were identified as having hopelessness. To evaluate nightmares, we used question 5-h of the Pittsburgh Sleep Quality Index (PSQI) [18]: “During the past month, how often have you had trouble sleeping because you had bad dreams?” Participants who chose the response options (1) (less than once a week), (2) (once or twice a week), or (3) (three or more times a week) for this question were identified as having nightmares, and those who chose (0) (not during the past month) as not having nightmares.

### Statistics

IBM SPSS Statistics 20 was used for analysis and the significance level was set at *p* < 0.05. Dunnett’s *t*-test was selected after analyzing the one-way ANOVA, and an ANCOVA was carried out with a Bonferroni correction. Significant differences were identified when the adjusted residual in the *χ*^2^ test was more than |1.96|. Multivariate logistic regression was carried out with the direct method and the confidence interval (CI) was set at 95 %.

### Ethical considerations

We ensured that none of the participants were pressured or harmed due to non-participation, and indicated to them that their response constituted their informed consent to participate. The subjects also received no reward for participation. Personal data were strictly monitored to maintain confidentiality and protect the privacy of the participants. This study was approved by the Kurume University Ethics Review Board and informed consent was obtained.

## Results

The scores (mean ± SD) for phase, quality, and quantity were 8.8 ± 3.3, 10.5 ± 3.0, and 8.1 ± 2.9. The All Good Sleep type was the most prevalent (19.2 %), and Inefficient + Short type was the second most prevalent (17.2 %). Owl + Inefficient type was the least prevalent (5.8 %). Table 2 shows the characteristics of each sleep type. With regard to age, we divided the participants into two groups based on the average and for ease of interpretation: 39 and younger versus 40 and older. Significantly high rates of participants under age 39 were found for both Owl type and Owl + Short type, while participants over age 40 were the most common among both Inefficient type and Inefficient + Short type. Many participants belonging to the All Good Sleep type were married, whereas many participants belonging to the All Poor Sleep type or Owl + Short type were single. In addition, there was a significant difference between the number of men and women only for the Short type. The symptoms of suicidal ideation, hopelessness, and nightmare were found significantly more frequent for All Poor Sleep compared to All Good Sleep. When All Good Sleep was set as the criterion of the Dunnett’s t-test for SDS scores (sleep item of the SDS was excluded), there were significant differences between all pairs except for All Good Sleep and Owl types. All Poor Sleep demonstrated the highest SDS score among all sleep types, and Inefficient + Short showed the second highest.

**Table 2:** Characteristics classified by 3-dimensional sleep scale (3DSS) scores

		Total	All Good Sleep	Owl	Inefficient	Short	Owl + Inefficient	Owl + Short	Inefficient + Short	All Poor Sleep
**Age**										
Under 39		274 (43.1)	48 (39.3)	44 (62.9)^a^	19 (31.1)^b^	27 (44.3)	15 (40.5)	46 (54.1)^a^	28 (25.7)^b^	47 (52.2)
Over 40		361 (56.9)	74 (60.7)	26 (37.1)^b^	42 (68.9)^a^	34 (55.7)	22 (59.5)	39 (45.9)^b^	81 (74.3)^a^	43 (47.8)
**Gender**										
Male		461 (72.6)	97 (79.5)	50 (71.4)	40 (65.6)	37 (60.7)^b^	30 (81.1)	56 (65.9)	83 (76.1)	68 (75.6)
Female		174 (27.4)	25 (20.5)	20 (28.6)	21 (34.4)	24 (39.3)^a^	7 (18.9)	29 (34.1)	26 (23.9)	22 (24.4)
**Marital status**										
Single		202 (31.8)	19 (15.6)^b^	24 (34.3)	16 (26.2)	11 (18.0)^b^	13 (35.1)	41 (48.2)^a^	27 (24.8)	51 (56.7)^a^
Married		406 (63.9)	97 (79.5)^a^	45 (64.3)	43 (70.5)	44 (72.1)	24 (64.9)	41 (48.2)^b^	75 (68.8)	37 (41.1)^b^
Other		27 (4.3)	6 (4.9)	1 (1.4)	2 (3.3)	6 (9.8)^a^	0 (0.0)	3 (3.5)	7 (6.4)	2 (2.2)
**Company (mainly)**										
Manufacturing		407 (64.1)	86 (70.5)	48 (68.6)	41 (67.3)	38 (62.3)	21 (56.7)	48 (56.4)	79 (72.5)	46 (51.1)
Service		228 (35.9)	36 (29.5)	22 (31.4)	20 (32.8)	23 (37.7)	16 (43.2)	37 (43.5)	30 (27.5)	44 (48.9)
**Suicidal ideation**										
No		516 (81.3)	116 (95.1)^a^	59 (84.3)	51 (83.6)	53 (86.9)	31 (83.8)	65 (76.5)	83 (76.1)	58 (64.4)^b^
Yes		119 (18.7)	6 (4.9)^b^	11 (15.7)	10 (16.4)	8 (13.1)	6 (16.2)	20 (23.5)	26 (23.9)	32 (35.6)^a^
**Hopelessness**										
No		465 (73.2)	106 (86.9)^a^	54 (77.1)	41 (67.2)	53 (86.9)^a^	25 (67.6)	58 (68.2)	74 (67.9)	54 (60.0)^b^
Yes		170 (26.8)	16 (13.1)^b^	16 (22.9)	20 (32.8)	8 (13.1)^b^	12 (32.4)	27 (31.8)	35 (32.1)	36 (40.0)^a^
**Nightmares**										
No		555 (87.4)	118 (96.7)^a^	64 (91.4)	52 (85.2)	57 (93.4)	35 (94.6)	73 (85.9)	85 (78.0)^b^	71 (78.9)^b^
Yes		80 (12.6)	4 (3.3)^b^	6 (8.6)	9 (14.8)	4 (6.6)	2 (5.4)	12 (14.1)	24 (22.0)^a^	19 (21.1)^a^
**SDS scores (Mean ± SD) without sleep item**		39.7 ± 7.3	34.9 ± 6.2^*^	37.5 ± 7.2	39.9 ± 7.2	38.0 ± 6.1	39.4 ± 5.9	40.7 ± 6.9	42.6 ± 6.9	44.4 ± 6.5

The overall SDS score (Mean ± SD) including a sleep item was 41.2 ± 7.6

*SDS* self-rating depression scale

^a^Significantly high rate by *χ* ^2^ test

^b^Significantly low rate by *χ* ^2^ test

* Significant differences between All Good Sleep and other sleep types, except Owl type, by Dunnett’s *t*-test

**Fig. 2:**
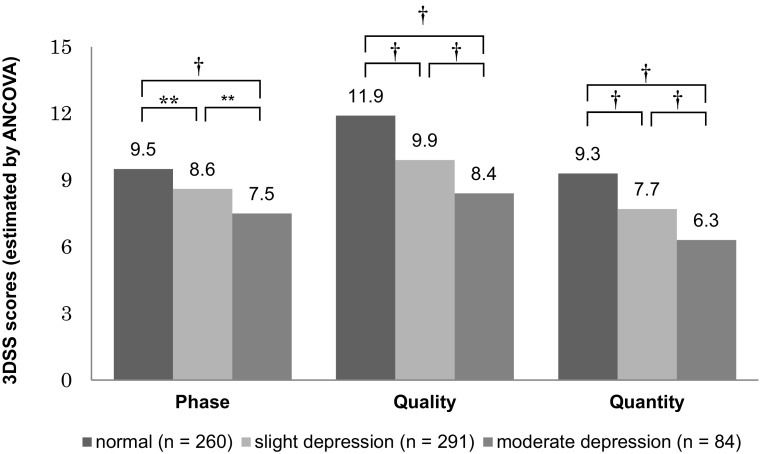
Estimated value of 3DSS scores and multiple comparisons by ANCOVA (adjusted for age, gender, marital status, and company). Participants were classified into three groups based on SDS scores (under 39 = normal, 40–49 = slight depression, over 50 = moderate depression). There were significant differences (**p < 0.01; ^†^ *p* < 0.001, Bonferroni correction) for all comparisons of 3DSS scores

Figure 2 shows 3DSS scores of the normal, slight depression, and moderate depression groups based on SDS scores with an ANCOVA test. There were significant differences for all comparisons of 3DSS scores. The normal group had the highest 3DSS scores (phase, quality, and quantity) among the three groups, and the moderate depression group had the lowest.

Table 3 shows the odds ratios for suicidal ideation for every sleep type with univariate or multivariate logistic regression. All Good Sleep, with a mean SDS score lower than that of any other sleep type, was set as the reference. The risk of suicidal ideation was significantly increased for every sleep type compared to All Good Sleep in model 2. After adjusting for hopelessness and nightmares, in model 3, Owl + Short, Inefficient + Short, and All Poor Sleep retained a significant risk of suicidal ideation. Overall, the greater the number of sleep problems, the higher the risk of suicidal ideation, with All Poor Sleep having the highest risk of suicidal ideation among the eight sleep types.

**Table 3:** Odds ratios and 95 % confidence intervals of suicidal ideation by sleep type

Sleep type	Model 1^a^		Model 2^b^		Model 3^c^	
	OR (95 % CI)	*p* value	OR (95 % CI)	*p* value	OR (95 % CI)	*p* value
All Good Sleep	Reference		Reference		Reference	
Owl	3.61 (1.27–10.2)	0.011*	3.14 (1.07–9.16)	0.037*	2.56 (0.86–7.65)	0.093
Inefficient	3.79 (1.31–11.0)	0.010*	3.80 (1.29–11.2)	0.016*	2.64 (0.86–8.05)	0.089
Short	2.92 (0.96–8.83)	0.049*	3.15 (1.02–9.73)	0.046*	3.01 (0.96–9.47)	0.059
Owl + Inefficient	3.74 (1.13–12.4)	0.023*	3.53 (1.04–12.0)	0.044*	2.67 (0.77–9.29)	0.122
Owl + Short	5.95 (2.27–15.6)	<0.001*	4.94 (1.84–13.3)	0.002*	3.76 (1.37–10.3)	0.010*
Inefficient + Short	6.06 (2.39–15.4)	<0.001*	6.12 (2.36–15.8)	<0.001*	4.49 (1.70–11.9)	0.002*
All Poor Sleep	10.7 (4.22–27.0)	<0.001*	8.83 (3.39–23.0)	<0.001*	6.02 (2.26–16.0)	<0.001*

^a^Unadjusted (crude) OR

^b^Adjusted for age, gender, marital status, and company (omnibus test, *p* < 0.05; Hosmer–Lemeshow test, *p* > 0.1)

^c^Adjusted for age, gender, marital status, company, hopelessness, and nightmares (omnibus test, *p* < 0.05; Hosmer–Lemeshow test, *p* > 0.1)

* Significant difference

## Discussion

This study demonstrated a relationship between sleep problems and depression, and in particular suicidal ideation or the process of suicide in Japan. Individuals living in a 24-h society suffer from problems with sleep phase as well as sleep quality or quantity; use of the 3DSS made it possible to measure these problems concurrently. Among the sleep types assessed, all sleep problems were related to suicidal ideation. Furthermore, this study provided data on young adults for whom the suicide rate has hardly improved. Of note, this study’s findings indicated that local or widespread sleep problems are associated with suicidal ideation, which depends on the severity of the sleep condition.

Significant differences were found for all comparisons of 3DSS scores based on SDS scores. Circadian rhythm sleep-wake disorder (CRSWD) or eveningness can cause depression or other distinct mental symptoms [9, 19, 20]. In addition, it is a common knowledge that both sleep quality and quantity are connected with depression [21–25]. Therefore, our results support findings of previous studies, and the 3DSS is considered to effectively assess sleep conditions.

Participants were classified into eight sleep types, which were found to exhibit different characteristics. Young people (under age 39) were likely to belong to the Owl or Owl + Short type, in concordance with the fact that eveningness was common among young people [9]. The Short type was frequently found among women, and may be related to Japanese culture. Japanese women, especially those over 30 years old, tend to have shorter sleep times than men, a trend opposite to that of many other countries [6]. Japanese men, and those who are older in particular, believe that housework is a woman’s responsibility; thus, their wives go to bed later and wake up earlier to do housework. Older participants (over age 40) were more likely to belong to the Inefficient or Inefficient + Short type. It is well known that aging decreases sleep quality [26–28]. However, we considered that participants classified into the Inefficient + Short type may be experiencing an abnormal condition, because sleep quality and quantity often change in the reverse direction of each other. That is, when one is decreased, the other is increased under normal conditions [29, 30]. The sleep of the Inefficient + Short type reflects a breakdown in normal homeostasis, reflected by the fact that this sleep type had the second highest SDS score among all types. The All Good Sleep type and All Poor Sleep type are direct opposites. Interestingly, the All Good Sleep type included a high proportion of married individuals, while the All Poor Sleep type had a high proportion of single individuals. As suggested, having a life partner worry about one’s health may inspire good sleep habits, while not having a partner may make one indifferent to their sleep habits.

The present study demonstrated that all sleep types that had one or more sleep problems were significantly associated with a risk of suicidal ideation. While degree of depression might be associated with suicide risk, there is a significant relationship between sleep (3DSS scores) and total depression (SDS scores). This means that depression may be a mediating variable between sleep and suicidal ideation. Mediating variables must not be added as covariates, because this may lead to false results or a weakened association. Thus, we selected specific depression symptoms (hopelessness and nightmares), which were related to suicidal ideation independent of insomnia [17], as covariates. After adjusting for these covariates, the sleep types that had one sleep problem and Owl + Inefficient did not retain a significant relationship. However, the sample size of these sleep types may not have been sufficient to conduct the analysis using the adjustment factors of Model 3. Since the *p* values were low, there may be significant relationships with a larger sample. Overall, the risk of suicidal ideation tended to increase when sleep problems were present in all models. The higher the number of sleep problems, the higher the risk of suicidal ideation compared to sleep types not indicative of problems (All Good Sleep). This indicates that a multiple risk factor syndrome may exist between sleep problems and suicidal ideation, such as metabolic syndrome. The interpersonal theory of suicide proposed by Joiner [31] and Van Orden [32] describes factors that are the roots of suicidal ideation, desire, and attempts, i.e., thwarted belongingness (I am alone), perceived burdensomeness (I am a burden), and capability for suicide. The presence of one of these factors may be related to suicidal ideation, the presence of two may lead to suicidal desire, and the presence of all three may be related to suicidal behavior. We hypothesized that sleep problems would be associated with the presence of these factors. A previous study reported that the frontal lobe, which is responsible for controlling reasoning, is more heavily damaged than any other parts of the brain by poor sleep [33]. Another study identified an overactive amygdala as a neural basis of depression and anxiety disorders caused by poor sleep [34]. These abnormal conditions of the brain amplify humans’ negative feelings such as delusions of guilt, which may cause thwarted belongingness or perceived burdensomeness. Furthermore, poor sleep causes individuals to make dangerous choices instead of safe choices [35]; thus, poor sleep could potentially increase capability for suicide. We believe that increased sleep problems may cause the presence or overlap of suicidal factors, which may increase the risk of suicidal ideation. This suggests biological validity of the results, because the findings were not contradictory to existing knowledge regarding epidemiology.

The present study supported that sleep problems (phase, quality, and quantity), which were assessed by the 3DSS, were related to suicidal ideation and that more of these problems meant a higher risk of suicidal ideation. Therefore, improvement of poor sleep may control or eliminate suicidal ideation. Since various sleep types involve different characteristics and backgrounds, we must guide sleep habits by referring to individual sleep type. The 3DSS could help us to assess sleep problems in this regard.

### Limitations

There are some limitations to this study. First, selection bias may have occurred because data were gathered from a few specific companies and may not reflect general trends, in particular, regarding the rate of suicidal ideation. In addition, this study was limited to day workers, while in Japan, the ratio of shift workers has been recently increasing. Second, since the judgment of suicidal ideation relied on only one question, which did not assess suicidal ideation directly, reliability may have been weaker compared to the use of an objective measure of suicidality. Therefore, the present study is considered to be a preliminary investigation. In addition, the analysis was not adjusted by family make-up, detailed occupation, business position, medical history, smoking, and drinking, which could affect sleep conditions or suicidal ideation. While there would be low quality of model fit if the model were adjusted for these factors, because of the presence of too many dummy variables, it is important to analyze with more detailed adjustment in future research. The causal relationship between suicidal ideation and sleep or other factors such as marriage was also not clarified, because the present study was cross-sectional in nature. Demonstrating the connection is more important than the causal relationship, because no differences have been shown between primary insomnia and secondary insomnia in International Classification of Sleep Disorders 3 (ICSD-3) [29]. In addition, further cohort studies are needed to establish the effect of correcting sleep on suicidal ideation using the 3DSS.

## Electronic supplementary material

Below is the link to the electronic supplementary material.

Supplementary material 1 (DOCX 21 kb)
