# Supplementary material for: Day workers suffering from a wider range of sleep problems are more likely to experience suicidality
Source: Sleep Biol Rhythms. 2016 Jun 23;14(4):369–76. doi: 10.1007/s41105-016-0067-5 (PMC5037152; doi:10.1007/s41105-016-0067-5)
Supplement: Supplementary file 1 — Supplementary material 1 (DOCX 21 kb) [file 41105_2016_67_MOESM1_ESM.docx]

|  | **Please choose the response that most applies to you within the past month**  **(not about special situations, but your usual habits)** | | | **Always** | **Often** | | **Rarely** | **Never** |
| --- | --- | --- | --- | --- | --- | --- | --- | --- |
| 1 | I sleep for less than 6 hours on weekdays. | | | [ 1 ] | [ 2 ] | | [ 3 ] | [ 4 ] |
| 2 | I cannot get enough sleep even though I want to. | | | [ 1 ] | [ 2 ] | | [ 3 ] | [ 4 ] |
| 3 | I go to bed at a fixed, regular time on weekdays and weekends. | | | [ 1 ] | [ 2 ] | | [ 3 ] | [ 4 ] |
| 4 | I wake up at a fixed, regular time on weekdays and weekends. | | | [ 1 ] | [ 2 ] | | [ 3 ] | [ 4 ] |
| 5 | I have a well-balanced breakfast every day. | | | [ 1 ] | [ 2 ] | | [ 3 ] | [ 4 ] |
| 6 | It takes me more than 30 minutes to fall asleep. | | | [ 1 ] | [ 2 ] | | [ 3 ] | [ 4 ] |
| 7 | I wake up more than twice a night. | | | [ 1 ] | [ 2 ] | | [ 3 ] | [ 4 ] |
| 8 | I wake up earlier than usual (over 2 hours), and cannot fall asleep again. | | | [ 1 ] | [ 2 ] | | [ 3 ] | [ 4 ] |
| 9 | I don’t sleep soundly. | | | [ 1 ] | [ 2 ] | | [ 3 ] | [ 4 ] |
| 10 | I worry that I cannot fall asleep. | | | [ 1 ] | [ 2 ] | | [ 3 ] | [ 4 ] |
| 11 | I don’t feel free from sleepiness or fatigue when I wake up. | | | [ 1 ] | [ 2 ] | | [ 3 ] | [ 4 ] |
| 12 | I feel sleepy not only in the afternoon, but also in the morning and/or evening. | | | [ 1 ] | [ 2 ] | | [ 3 ] | [ 4 ] |
| 13 | I often doze off. | | | [ 1 ] | [ 2 ] | | [ 3 ] | [ 4 ] |
| 14 | “Morningness” is better suited to me than “Eveningness.” | | | [ 1 ] | [ 2 ] | | [ 3 ] | [ 4 ] |
| 15 | What time do you wake up on weekdays?  [ 1 ]→about 6:00 a.m. or earlier than 6:00 a.m.  [ 2 ]→about 6:30 a.m.  [ 3 ]→about 7:00 a.m.  [ 4 ]→later than 7:00 a.m. | | | [ 1 ] | [ 2 ] | | [ 3 ] | [ 4 ] |
| **Categories** | | **Item** | **Scoring** | | | **Range**  **(poor–good)** | | |
| Sleep Phase | | 3, 4, 5, 14, 15 | [ 1 ] → 3, [ 2 ] → 2, [ 3 ] → 1, [ 4 ] → 0 | | | 0–15 | | |
| Sleep Quality | | 6, 7, 8, 9, 10 | [ 1 ] → 0, [ 2 ] → 1, [ 3 ] → 2, [ 4 ] → 3 | | | 0–15 | | |
| Sleep Quantity | | 1, 2, 11, 12, 13 | [ 1 ] → 0, [ 2 ] → 1, [ 3 ] → 2, [ 4 ] → 3 | | | 0–15 | | |

**Supplementary Material.** 3 Dimensional Sleep Scale (translated from Japanese into English) and scoring method.
